# Alcohol Use during COVID-19 Pandemic: A Cross-Sectional Survey among Healthcare and Office Workers in Italy

**DOI:** 10.3390/ijerph191912587

**Published:** 2022-10-02

**Authors:** Fabrizio Cedrone, Giuseppe Buomprisco, Mucci Nicola, Giuseppe La Torre, Hector Nieto, Roberto Perri, Vincenzo Montagna, Emilio Greco, Simone De Sio

**Affiliations:** 1 Hospital Management, Local Health Authority of Pescara, 65122 Pescara, Italy; 2 R.U. of Occupational Medicine, “Sapienza” University of Rome, 00185 Rome, Italy; 3 Department of Experimental and Clinical Medicine, University of Florence, 50134 Florence, Italy; 4 Department of Public Health and Infectious Diseases, “Sapienza” University of Rome, 00185 Rome, Italy; 5 Occupational Medicine School, University of Business and Social Sciences, Buenos Aires C1061 ABA, Argentina; 6 Section of Hygiene, Department of Biomedical Science and Public Health, School of Medicine, Università Politecnica delle Marche, 60126 Ancona, Italy; 7 Faculty of Innovative Technologies for Digital Communication, Link Campus University, Via del Casale di San Pio V 44, 00165 Roma, Italy

**Keywords:** alcohol, COVID-19, AUDIT-C, alcohol use disorders, harmful alcohol consumption, lockdown, workers

## Abstract

Background: The aim of our study of a sample of Italian healthcare (HCWs) and office workers (OWs) carried out during the pandemic period was to understand alcohol consumption patterns during the COVID-19 pandemic. Methods: A web-based cross-sectional survey based on Google Forms was developed. Harmful alcohol use was assessed through a validated questionnaire (AUDIT-C). Three multivariate logistic regression models were implemented for the overall sample of HCWs and OWs. The presence of harmful alcohol consumption (AUDIT-C score) was considered as a dependent variable. Results: A total of 1745 workers answered the survey. A lower risk of harmful drinking behavior among men overall and in both working groups was found (aOR 0.42, CI 95% 0.33–0.53), but also for both HCWs (aOR 0.62, CI 95% 0.46–0.84) and OWs (aOR 0.17, CI 95% 0.11–0.27). Comparing OWs and HCWs, we found a higher risk of harmful drinking in the first group (aOR 1.62, CI 95% 1.20–2.18). Conclusions: The results of the survey indicate that unhealthy behaviors were elevated during the pandemic. It is urgent to implement company policies managed by an occupational doctor to raise workers’ awareness of alcohol-related dangers and provide educational tools that have the task of preventing the damage caused by alcohol.

## 1. Introduction

Italy was the first European country severely hit by the COVID-19 pandemic [1]. Quarantines and lockdowns were the principal measures able to contain the spread of the virus, thus forcing people to fit their needs with isolation protocols. This social distancing caused many lifestyle changes affecting both working and private life. Worktime became more stressful, with much more attention paid to sanitizing measures and safety procedures. Healthcare workers were the most psychologically affected: working on the front line to fight the virus spreading and trying to protect colleagues and family from contagion while caring for life-threatened patients were very stressful tasks [2]. The consequences on mental health included adverse psychological responses such as anxiety and depression [3]. As demonstrated by a recent systematic review, mood and anxiety disorders were particularly prevalent in substance-use treatment clients [4]. Most office workers were able to continue working thanks to telework. That kind of working arrangement, however, forced them to stay at home, enhancing social isolation and family conflicts.

Pandemic rules led to less social support and less recreative time (sports and entertainment), which could lead to self-harmful behaviors such as heavy drinking and changes in eating habits [5]. Psychological distress, enhanced by the interaction of financial difficulties, social isolation, and uncertainty about the future during and after the COVID-19 pandemic, could worsen patterns of alcohol use and increase its attributable harm [6]. With these premises, alcohol policy experts have demonstrated how a crisis like the COVID-19 pandemic might impact alcohol consumption and suggest that this association is due to the use of alcohol and other substances in order to cope with such stressful conditions [7]. This was also observed during the SARS outbreak of 2003 [8].

Alcohol use, besides being a significant risk factor for mental health disorders, is a major public health concern [9]. Recent findings agree that the stress and isolation experienced during the COVID-19 pandemic could act as a trigger for alcohol abuse and could lead to an increase in the prevalence of alcohol use disorders (AUDs) and alcohol-related health problems [10,11,12]. Moreover, the patterns of alcohol consumption varied during the phases of lockdown: first, there was an increase in alcohol-related emergencies (alcohol withdrawal, related suicides, and methanol toxicity), then changes in the quantity of drinking (i.e., binge/heavy drinking) were observed during the lockdown, as well as relapses post-lockdown [13].

Linked to this topic, some of the authors who worked on this paper assessed how working status can predict alcohol consumption, comparing alcohol-related biomarker levels in office workers and unemployed people, finding office work as a negative predictor of pathological alcohol-linked biomarker levels [14].

The aim of our study, carried out during the COVID-19 pandemic, was to understand which socio-demographic, work, and lifestyle variables were linked to harmful alcohol use among a sample of Italian healthcare and office workers.

## 2. Materials and Methods

A web-based cross-sectional survey based on Google Forms was developed. Participation, voluntary and anonymous, was available across the first wave of the COVID-19 pandemic and subsequent lockdown period that started in Italy on March 9 2020 and ended in late May 2020. This cross-sectional study examined a large convenience sample throughout Italy. Participants were recruited through the mailing lists of the Occupational Medicine Research Unit of Sapienza University of Rome. One was made of doctors, considered as a group healthcare workers (HCWs), and one of office workers (OWs). A total of 2222 emails were sent.

**Dependent Variable**

The Alcohol Use Disorders Identification Test-Concise (AUDIT-C) scale [15] is a validated, short, modified version of the 10-item AUDIT instrument. It has been successfully used in the past to assess alcohol consumption habits both in the general and in the working population, healthcare workers included [16,17,18]. That tool has also been adopted during the confinement measures in response to COVID-19 to monitor alcohol habits among the general population in the US, and a high increase in harmful alcohol consumption was observed over the first six months of the pandemic [19].

The AUDIT-C is scored on a scale of 0–12 (0 indicates no alcohol use). A score of 4 or higher for men and 3 or higher for women indicates harmful alcohol consumption. Generally, the higher the AUDIT-C score, the more likely it is that drinking is affecting one’s health and safety. Respondents with a score lower than 4 for men and 3 for women are considered low-risk alcohol consumers.

The dependent variable was constructed by dichotomizing the sample between who reported an alcohol consumption risk and who had not.

**Independent variables**

An ad-hoc questionnaire was developed to investigate:

- *Socio-demographic variables*: gender, age, cohabitants (alone, family, mates);
- *Working variables*: contract type (permanent, fixed-term), job seniority (years), shift work (yes, no), job demands during the pandemic (lower, same, higher);
- *Lifestyle variables*: smoking (yes, no), the number of daily smoked cigarettes, changes in eating habits (yes, no), changes in food intake (less, more, same), drinking more alcohol than before the pandemic (yes, no).

### Statistical Analysis

Quantitative variables were expressed as the median and the interquartile range (IQR); qualitative variables were indicated as frequency and percentage. Univariate analysis, including chi-square for categorical variables and nonparametric tests (Mann–Whitney) for skewed quantitative variables, was conducted to assess differences between groups of descriptive variables and the outcome of the questionnaires (dichotomous) for the overall sample of HCWs and OWs. Three multivariate logistic regression models were implemented for the overall sample as follows: The presence of harmful alcohol consumption (AUDIT-C score) was considered a dependent variable and each one of the available factors at the baseline evaluation as independent variables. Statistical significance was set at a two-sided *p* < 0.05. All analyses were performed using STATA^®^ software (version 14; Stata Corp LP, College Station, TX, USA).

## 3. Results

### 3.1. Univariate Analysis

A total of 1745 workers (1170 HCWs and 575 OWs) answered the survey (response rate of 78.6%). According to the results of the AUDIT-C questionnaire, 466 of them reported a harmful level of alcohol consumption (317 HCWs and 149 OWs) while the remaining 1279 were deemed to not be at risk. The results are summarized in Table 1.

In the total sample, low-risk consumers consisted of 682 males and 597 females (53.32% and 46.68%), while harmful ones comprised 165 males and 301 females (35.41% and 64.59%). Among HCWs, low-risk consumers consisted of 385 males and 468 females (45.13% and 54.87%) and harmful ones comprised 114 males and 203 females (35.96% and 64.04%). In the OW group, low-risk consumers consisted of 297 males and 129 females (69.72% and 30.28%) and harmful ones comprised 51 males and 98 females (34.63% and 65.77%). 

Median age was 42 years (IQR 34–52) among low-risk consumers, while it was 40 years (IQR 33–52) among harmful ones.

A higher percentage of workers had a permanent contract work type in both groups: overall there were 1300 permanent workers and 445 with a fixed-term contract, equally distributed between the two groups. Median job seniority was similar in all groups: 10 years (IQR 4–20) among low-risk consumers and a little lower among harmful-drinking HCWs (9 years, IQR 3–20) and OWs (7 years, IQR 3–15) consumers.

Smoking habits were largely found in each group: 54.27% of respondents were smokers. A half of the low-risk alcohol consumers were smokers (50.82%); that percentage was higher among harmful alcohol consumers (63.73%). These proportions were similar in the two study groups.

The participants were also asked about drinking habit changes during the pandemic period: 57.31% declared they had not increased alcohol consumption. Most HCWs increased alcohol consumption in both low-risk and harmful consumer groups (56.86% and 51.42% respectively), while most of the OWs had not increased alcohol consumption in either group (low-risk consumers 89.67% and harmful consumers 64.43%).

### 3.2. Multivariable Logistic Regression

The results of the bivariate analysis are summarized in Table 2.

Gender was an important variable overall for both HCWs and OWs. A lower risk of harmful drinking behavior among men overall and in both working groups was found (aOR 0.42, CI 95% 0.33–0.53, *p*-value < 0.001), but also for both HCWs (aOR 0.62, CI 95% 0.46–0.84, *p*-value 0.002) and OWs (aOR 0.17, CI 95% 0.11–0.27, *p*-value < 0.001).

Comparing OWs and HCWs, we found a higher risk of harmful drinking in the first group (aOR 1.62, CI95% 1.20–2.18) with a *p*-value of 0.001. 

Smoking was associated with harmful drinking in all groups, with a higher risk for HCWs (aOR 3.43, CI 95% 2.30–5.12, *p*-value < 0.001) and a little lower for OWs (aOR 1.85, CI 95% 1.16–2.97, *p*-value 0.010). Overall, we found smoking habit as a risk factor (aOR 2.14, CI 95% 1.62–2.84, *p*-value of 0.036).

## 4. Discussion

Many authors investigated the impact of the COVID-19 pandemic on alcohol use across Europe. Matone et al. conducted a cross-sectional online survey among more than 40,000 adults in 21 European countries during the first wave of the pandemic in early 2020. The authors reported that subjects with a risky or hazardous use of alcohol increased both drinking quantity and frequency [20]. The same conclusion about people with pre-existing high drinking levels/alcohol use disorders was reached by a large meta-analysis published by Kilian et al. The authors, however, also found that more people generally reduced their alcohol use in Europe than increased it since the onset of the pandemic [21]. 

In Italy, other authors have looked at the effect of the pandemic on alcohol use in the general population using the Audit-C questionnaire. They found that about 18% of the respondents increased their alcohol consumption and this showed a significant correlation with the anxiety and fear experienced [22]. Similar conclusions were reached by another study of the impact of the COVID-19 lockdown on mental health. Researchers found that having a hazardous alcohol-drinking habit before the national lockdown was associated with an increase in anxiety symptoms [23]. 

The results of our survey provides some food for thought.

In recent years, there has been a reduction in the gap between men and women both for characteristics of alcohol consumption (e.g., prevalence, frequency, and amount) and problematic drinking (e.g., binge drinking and early-onset drinking) [24]. Regarding gender, a large study conducted in the USA starting from the consideration that women in the United States consume less alcohol and suffer less alcohol-related harm than males showed that the differences in consumption between 2012 and 2022 were reduced [25]. A recent study conducted during COVID-19 showed that the percentage of females with risky consumption was higher than that of males, both before and during confinement [26]. Our data strongly support this tendency, but that phenomenon is very worrying and must be monitored, given the greater susceptibility of women to alcohol-related harm [27]. Previous research has demonstrated that female workers perceive a greater amount of work-related stress than their male colleagues [28].

During the COVID-19 emergency, due to the increased stress for both health practitioners and office workers, addictive behaviors could represent a coping mechanism: SARS-CoV-2’s spread caused heavier workloads in health facilities, and, at the same time, a very large amount of healthcare workers had to increase their work commitments in order to improve health system compliance. Office workers, on the other hand, were locked up at home and, in addition to having to carry out their work in an unsuitable environment, they had to deal with changed family dynamics (children at home, isolation, lack of recreational/sports activities, etc.). Those changes probably led to the search for mechanisms to compensate for the stress that was created, for example alcohol consumption. That is consistent with a recent study in the US in which the participants who reported being stressed by the pandemic consumed more drinks over a greater number of days [29].

The differences found by comparing OWs and HCWs may be caused by dissimilar educational paths, with healthcare professionals being much more aware of heavy alcohol consumption risks. In Italy, since the beginning of the COVID-19 spread, telework has been a largely diffused solution to reduce risky contact in workplaces: this further isolation from coworkers could affect the stressful perception linked to the pandemic [2,30,31,32]. That agrees with other research that shows that having effective social support is one of the most significant correlates of well-being and is assumed to positively impact health and guard against distress [33]. Working at home reduces interaction with colleagues and, thus, the opportunity to have work-related social support, a recognized important coping strategy [34]. Very recent data support that consideration because increased alcohol consumption during lockdown was reported among people working at home [35].

The result we obtained regarding smoking was expected. It cannot be considered a consequence of the COVID-19 pandemic but, instead, as a well-known complementary relationship between these two habits (smoking and drinking alcohol) [36]. 

This study, based on a web survey, has a few limitations. First of all, cross-sectional studies are limited in assessing the temporal relationship between exposure and outcome. Secondly, because of the method of recruiting participants, the raw results could lack generalization and could not be immune from possible response bias. The most important limitation of our study is the self-reported nature of our data. At the same time, the online questionnaire did not allow respondents to ask for help if any part of the survey was not understood. Therefore, it should be considered that a web-based survey is the most rapid and reliable method to collect data in a short period, in particular, if social interactions are forbidden (i.e., lockdown). The strengths of the research are the use of a validated assessment tool and the good study sample, which can reflect the entire population of workers studied. In addition, the statistical analysis enforces the validity of the results. Despite this, recall bias could occur because of the self-reporting nature of the assessment tools.

## 5. Conclusions

The results of the survey indicate that unhealthy behaviors increased among the respondents during the pandemic. Regarding drinking habits and COVID-19 lockdown, the actual evidence is conflicting. Recent findings of an Italian survey report a reduction in alcohol intake, probably due to reduced social/recreational drinking. Other authors reported an increase in alcohol consumption in approximately 14% of participants, and this corresponds quite well with our data. It is urgent to implement company policies with health promotion programs (managed by an occupational doctor) through seminars, focus groups, and personal interviews, where workers are exposed to education about the harmful effects of alcohol abuse on health, in order to raise workers’ awareness of alcohol-related dangers and provide educational tools that have the task of preventing the damage caused by alcohol.

## Figures and Tables

**Table 1:** Results of the univariate analysis.

	Overall				HCWs			OWs		
		Low-Risk Drinking	Harmful Drinking	*p*-Value	Low-Risk Drinking	Harmful Drinking	*p*-Value	Low-Risk Drinking	Harmful Drinking	*p*-Value
	N (%)	N (%)	N (%)		N (%)	N (%)		N (%)	N (%)	
**Total**	1745	1279	466		853	317		426	149	
**Sex**										
**Male**	847 (48.54)	682 (53.32)	165 (35.41)	<0.001	385 (45.13)	114 (35.96)	0.005	297 (69.72)	51 (34.23)	<0.001
**Female**	898 (51.46)	597 (46.68)	301 (64.59)		468 (54.87)	203 (64.04)		129 (30.28)	98 (65.77)	
Age **	42 (34–52)	42 (34–52)	40 (33–52)	0.108	42 (35–55)	42 (34–55)	0.391	41 (33–49)	39 (31–48)	0.055
**Contract type**										
**Permanent**	1300 (74.50)	970 (75.84)	330 (70.82)	0.033	614 (71.98)	219 (69.09)	0.331	356 (83.57)	111 (74.50)	0.015
**Fixed-term**	445 (25.50)	309 (24.16)	136 (29.18)		239 (28.02)	98 (30.91)		70 (16.43)	38 (25.50)	
Job seniority **	10 (4–20)	10 (4–20)	9 (3–20)	0.033	10 (4–20)	10 (3–22)	0.214	10 (4–20)	7 (3–15)	0.137
**Shift work**										
**No**	1400 (80.23)	1035 (80.92)	365 (78.33)	0.228	671 (78.66)	239 (75.39)	0.232	364 (85.45)	126 (84.56)	0.794
**Yes**	345 (19.77)	244 (19.08)	101 (21.67)		182 (21.34)	78 (24.61)		62 (14.55)	23 (15.44)	
**Job demands**										
**Lower**	527 (30.20)	379 (29.63)	148 (31.76)	0.553	272 (31.89)	111 (35.02)	0.500	107 (25.12)	37 (24.83)	0.196
**Same**	452 (25.90)	339 (26.51)	113 (24.25)		140 (16.41)	54 (17.03)		199 (46.71)	59 (39.60)	
**Higher**	766 (43.90)	561 (43.86)	205 (43.99)		441 (51.70)	152 (47.95)		120 (28.17)	53 (35.57)	
**Cohabitants**										
**Alone**	261 (14.96)	197 (15.40)	64 (13.73)	0.117	135 (15.83)	39 (12.30)	0.302 *	62 (14.55)	25 (16.78)	0.008
**Family**	1435 (82.23)	1052 (82.25)	383 (82.19)		702 (82.30)	273 (86.12)		350 (82.16)	110 (73.83)	
**Roommates**	49 (2.81)	30 (2.35)	19 (4.08)		16 (1.88)	5 (1.58)		14 (3.29)	14 (9.40)	
**Smoking**										
**No**	798 (45.73)	629 (49.18)	169 (36.27)	<0.001	301 (35.29)	77 (24.29)	<0.001	328 (77.00)	92 (61.74)	<0.001
**Yes**	947 (54.27)	650 (50.82)	297 (63.73)		552 (64.71)	240 (75.71)		98 (23.00)	57 (38.26)	
Daily n. cigarettes **	10 (5–15)	10 (5–15)	10 (4–15)	0.300	10 (5–15)	10 (4–15)	0.344	10 (5–15)	10 (5–15)	0.690
**Changing eating habits**										
**No**	996 (57.08)	726 (56.76)	270 (57.94)	0.660	549 (64.36)	202 (63.72)	0.840	177 (41.55)	68 (45.64)	0.385
**Yes**	749 (42.92)	553 (43.24)	196 (42.06)		304 (35.64)	115 (36.28)		249 (58.45)	81 (54.36)	
**Food intake**										
**Less**	543 (31.46)	411 (32.13)	132 (28.33)	0.149	272 (31.89)	91 (28.71)	0.468	139 (32.63)	41 (27.52)	0.228
**More**	596 (34.53)	421 (32.92)	175 (37.55)		263 (30.83)	108 (34.07)		158 (37.09)	67 (44.97)	
**Same**	587 (34.01)	447 (34.95)	159 (34.12)		318 (37.28)	118 (37.22)		129 (30.28)	41 (27.52)	
**Drinking more alcohol than before**										
**No**	1000 (57.31)	750 (58.64)	250 (53.65)	0.062	368 (43.14)	154 (48.58)	0.096	382 (89.67)	96 (64.43)	<0.001
**Yes**	745 (42.69)	529 (41.36)	216 (46.35)		485 (56.86)	163 (51.42)		44 (10.33)	53 (35.57)	
AUDIT-C score **	2 (1–3)	1 (0–2)	4 (3–5)	<0.001	1 (0–2)	4 (3–5)	<0.001	2 (0–2)	4 (3–5)	<0.001

* Fisher exact. ** Median (IQR).

**Table 2:** Multivariable logistic regression model results, dependent variable (harmful alcohol use).

	Overall		HCWs		OWs	
	aOR (95%CI)	*p*-Value	aOR (95%CI)	*p*-Value	aOR (95%CI)	*p*-Value
**Male vs. female**	0.42 (0.33–0.53)	<0.001	0.62 (0.46–0.84)	0.002	0.17 (0.11–0.27)	<0.001
Age *	0.99 (0.97–1.01)	0.373	0.99 (0.96–1.01)	0.471	1.00 (0.97–1.03)	0.932
**Contract type**						
**Fixed-term vs.** **Permanent**	1.28 (0.97–1.62)	0.056	1.23 (0.90–1.69)	0.192	1.95 (1.11–3.42)	0.019
**OWs vs. HCWs**	1.62 (1.20–2.18)	0.001	-	-	-	-
Job seniority *	1.01 (0.99–1.02)	0.457	1.01 (0.98–1.03)	0.438	0.98 (0.95–1.01)	0.436
**Shift work**						
**Yes vs. no**	1.21 (0.92–1.61)	0.169	1.27 (0.90–1.79)	0.169	0.83(0.45–1.52)	0.562
**Job demands**						
**Lower**	Ref.		Ref.		Ref.	
**Same**	0.91 (0.66–1.24)	0.548	0.98 (0.66–1.47)	0.943	1.13 (0.64–1.98)	0.668
**Higher**	1.03 (0.78–1.36)	0.816	0.88 (0.63–1.22)	0.459	1.85 (1.01–3.37)	0.044
**Cohabitants**						
**Alone**	Ref.		Ref.		Ref.	
**Family**	1.17 (0.85–1.61)	0.317	1.35 (0.91–2.01)	0.132	0.98 (0.53–1.81)	0.969
**Roommates**	1.65 (0.83–3.27)	0.151	0.81 (0.26–2.52)	0.724	1.84 (0.65–5.19)	0.248
**Smoking**						
**Yes vs. no**	2.14 (1.62–2.84)	0.036	3.43 (2.30–5.12)	<0.001	1.85 (1.16–2.97)	0.010
**Food intake**						
**Less**	Ref.		Ref.		Ref.	
**Same**	1.19 (0.91–1.58)	0.200	1.14 (0.82–1.60)	0.417	1.24 (0.70–2.19)	0.458
**More**	1.33 (1.01–1.76)	0.036	1.28 (0.91–1.79)	0.151	1.67 (0.98–2.85)	0.058
**Drinking more than before**						
**Yes vs. no**	1.01 (0.77–1.34)	0.925	0.41 (0.29–0.59)	<0.001	5.37 (3.15–9.15)	<0.001

* Years. aOR: adjusted Odds Ratio. CI: Confidence Interval.

## Data Availability

Data available under request.
